# IL-10 ameliorates TNF-α induced meniscus degeneration in mature meniscal tissue in vitro

**DOI:** 10.1186/s12891-017-1561-x

**Published:** 2017-05-16

**Authors:** P. Behrendt, K. Häfelein, A. Preusse-Prange, A. Bayer, A. Seekamp, B. Kurz

**Affiliations:** 1 Department of Orthopaedics and Trauma Surgery, University Medical Center Schleswig-Holstein, Campus Kiel, Kiel, Germany; 2 Institute of Anatomy, Christian Albrechts-University, Kiel, Germany; 3 Department of Cardiovascular Surgery, University Medical Center Schleswig-Holstein, Campus Kiel, Kiel, Germany

**Keywords:** Inflammation, Interleukin 10, Meniscus, TNF-α

## Abstract

**Background:**

Joint inflammation causes meniscus degeneration and can exacerbate post-traumatic meniscus injuries by extracellular matrix degradation, cellular de-differentiation and cell death. The aim of this study was to examine whether anti-inflammatory interleukin-10 exerts protective effects in an in vitro model of TNF-α-induced meniscus degeneration.

**Methods:**

Meniscus tissue was harvested from the knees of adult cows. After 24 h of equilibrium explants were simultaneously treated with bovine TNF-α and IL-10. After an incubation time of 72 h cell death was measured histomorphometrically (nuclear blebbing, NB) and release of glycosaminoglycans (GAG, DMMB assay) and nitric oxide (NO, Griess-reagent) were analysed. Transcription levels (mRNA) of matrix degrading enzymes, collagen type X (COL10A1) and nitric oxide synthetase 2 (NOS2) were measured by quantitative real time PCR. TNF-α-dependent formation of the aggrecanase-specific aggrecan neoepitope NITEGE was visualised by immunostaining. Differences between groups were calculated using a one-way ANOVA with a Bonferroni post hoc test.

**Results:**

Administration of IL-10 significantly prevented the TNF-α-related cell death (P .001), release of NO (P .003) and NOS2 expression (P .04). Release of GAG fragments (P .001), NITEGE formation and expression of MMP3 (P .007), -13 (P .02) and ADAMTS4 (P .001) were significantly reduced. The TNF-α-dependent increase in COL10A1 expression was also antagonized by IL-10 (P .02).

**Conclusion:**

IL-10 prevented crucial mechanisms of meniscal degeneration induced by a key cytokine of OA, TNF-α. Administration of IL-10 might improve the biological regeneration and provide a treatment approach in degenerative meniscus injuries and in conditions of post-traumatic sports injuries.

## Background

Joint diseases apply to approximately 15% of world’s population. In a healthy knee joint, the meniscus integrity is a prerequisite for the joints kinematics and its load balancing. In the case of a partial meniscectomy a 10% reduction in meniscal contact area was demonstrated, which produced a 65% increase in peak joint-contact stress [1], leading to an early development of osteoarthritis (OA) [2]. Hence, post-traumatic and degenerative damages of the meniscus are well accepted as major predisposing factors for OA and post-traumatic OA (PTOA) [3, 4].

Degenerative meniscal tears predominantly occur in the postero-medial meniscus horn because of the highest force uptake during weight bearing in this area [5]. Meniscus degeneration takes place as a biomechanical adaptation to elevated loads and is accompanied by fibrocartilage formation and calcification in the medial meniscus posterior horn, which in turn is associated with the degree of the tear [6]. Both mechanisms are known to impair the resistance to tension and act as adaptation to increased mechanical overload [7, 8]. Histopathological characteristics of fibrocartilage metaplasia include joint inflammation, matrix remodeling by matrix metalloproteinases, collagen disruption and calcification. Du et al. demonstrated that mechanical overload induces meniscus hypertrophy and therefore called for anti-hypertrophic drugs to prevent meniscus degeneration and subsequent PTOA [9]. In addition, traumatic injuries of the meniscus lead to raised levels of proinflammatory cytokines, such as TNF-α [10]. In vitro models have pointed out the relevance of an inflammatory reaction induced by mechanical overload, which subsequently caused matrix degeneration and induction of cell apoptosis [11, 12]. In terms of traumatic anterior cruciate ligament rupture, meniscal tear and early OA, this inflammation was demonstrated acute after the injury [13]. Inflammatory events occured after meniscal injury regardless of preexisting osteoarthritic changes in the joint [14]. Proinflammatory cytokines have been shown to suppress matrix biosynthesis and increase enzymatic matrix degradation. TNF-α is known to induce cellular apoptosis by direct stimulation of death receptors. In addition, TNF-α induces matrix-metalloproteinases and aggrecanases as well as nitric oxide (NO) production, which in turn cause cell death and matrix degeneration [15–17]. The intrinsic repair capacity of the meniscus tissue is significantly inhibited at high concentrations of TNF-α (10 ng/ml) [18].

Yet, therapeutically augmentation strategies to promote the meniscus regeneration are still a field of study. Current interest is growing in the use of tissue growth factors to facilitate meniscus regeneration and restore a functional meniscus. The administration of growth factors may be a supplementary option in terms of surgical meniscus repair and usage of exogenous materials for meniscus regeneration. In this context, various studies have shown protective effects of anti-inflammatory interleukin 10 (IL-10) on a wide range of tissues including articular cartilage and synovialocytes [19]. Interleukin 10 is a potent anti-inflammatory and immune-regulatory cytokine. Apart from its immunological properties IL-10 is involved in processes such as connective tissue extracellular matrix remodeling and matrix homeostasis [19]. It has been shown to antagonize matrix degeneration, induced by proinflammatory cytokines such as TNF-α in experimental models of OA and rheumatic arthritis [19, 20]. IL-10 restored inhibition of proteoglycan synthesis in cartilage explants of joints with end-stage haemophilic arthropathy [21]. In addition, IL-10 seems to have anti-apoptotic effects since it was shown to inhibit TNF-α induced caspase activities and restored the impaired bax/bcl-2 ratio in articular chondrocytes [22]. Meniscus degeneration and development of OA are closely interactive. The aim of this study was to test the hypothesis that IL-10 treatment will lead to a decrease of inflammation-induced loss of tissue viability and matrix degeneration in an in vitro model of bovine meniscal tissue.

## Method

### Isolation and culture of mature meniscal explants

This study was conducted using explants from five donor animals. Approval was obtained from the institutional ethics committee prior to performing the study. Meniscus explants were isolated from the articular cavity of knee joints from 16 to 24 months old cows procured from a local abattoir authorized by the relevant meat inspectors as described previously [23, 24]. Using a 10 mm biopsy punch full thickness tissue cylinders were harvested from the red-white zone of the meniscus. Tissue cylinders were loaded in a sample holder and trimmed to yield 1 mm thick disks. Thereby the specimens lost the original meniscal surface. Four to five smaller explants (3 mm in diameter × 1 mm thickness) were harvested from each disk using a 3 mm biopsy punch (Hebu-medical, Tuttlingen, Germany). Weight of explants were measured. Explants with appropriate thickness were isolated, randomly distributed (for one individual experiment) and thereafter cultured separately in 250 μl medium in 96-well plates. An equilibration was performed for 24 h at 37 °C in an atmosphere of 5% CO_2_ in culture medium (low-glucose Dulbecco’s modified Eagle’s medium (Biochrom, Berlin, Germany) supplemented with 10 mM HEPES buffer (Biochrom, Berlin, Germany), 0.4 mM proline (Sigma-Aldrich, Darmstadt, Germany), 50 μg vitamin c, 100 units/ml of penicillin G, 100 mg/ml of streptomycin, and 0.25 mg/ml of amphotericin B (PAA Laboratories, Pasching, Germany)).

### Incubation with TNF-α and stimulation with IL-10

Treatment was initiated after 24 h of equilibration by renewing the culture medium containing bovine TNF-α (R&D systems, Wiesbaden-Nordenstadt Germany; 10 and 100 ng/ml) with or without bovine IL-10 (Kingfisher Biotech, Saint-Paul, MN, USA) in different concentrations (10 pg/ml – 20 ng/ml). An alignment analysis of the protein sequences of bovine and human IL-10 revealed a homology of 77.52%. Treatment was performed for 72 h at 37 °C in an atmosphere of 5% CO_2_. After that supernatants were frozen and explants were collected for real time qPCR or processed to histological analysis.

### Histological detection of cell death and immunohistochemistry

In order to visualize cells with nuclear bleebing indicating apoptotic cell death [25], explants were fixed overnight using 4% paraformaldehyde (in PBS), embedded in Paraplast, sectioned (7 μm) and stained with Mayer’s haematoxylin as described previously [24]. Images were taken using a Zeiss Axiophot microscope (Zeiss, Wetzlar, Germany) and the number of apoptotic cells was quantified [in %] in relation to the total number of cells per optical field by a blinded investigator. While margins of the sections (150 μm depth) were excluded, the value for each explant was calculated from three different areas.

For immunohistochemical visualisation of aggrecanase-specific aggrecan neoepitope NITEGE formation, immobilized explants were incubated for 2.5 min in a digester at 100 °C (in 0.01 M citric acid, pH 6.0) and thereafter incubated overnight at 4 °C with the primary antibody (Rabbit-IgG-Aggrecan Neo Polyclonal Antibody PA-1746; 1:50 dilution in 1% BSA; Thermofisher Scientific, Waltham, MA, USA) as described previously [24]. Sections were rinsed in Tris-NaCl and incubated with the secondary antibody AlexaFluor 488 goat anti-rabbit IgG (1:700; Invitrogen, Carlsbad, CA, USA) for one hour at room temperature. Additional nuclear staining was performed with bisbenzimide (Sigma-Aldrich, St. Louis, MO, USA). Images were taken using the Apotome (ZEISS, Jena, Germany) fluorescence microscope.

### Measurement of glycosaminoglycans (GAG) and nitric oxide (NO)

Release of GAGs in the supernatant was quantified by dimethlmethylene blue dye (DMMB) assay at a wavelength of 525 nm (Photometer Ultraspec II, Biochrom, Cambridge, UK) using shark chondroitin-sulfate as standard [26]. Values are given as μg GAG/mg explant wet weight.

Release of NO in the supernatant was quantified by Griess reagent (1% sulphanilamide and 0.1% N-(1-naphtyl)-ethylene diamine-dihydro-chloride in 5% phosphoric acid (H_3_O_4_P), Sigma-Aldrich, St. Louis, MO, USA) using sodium nitrite (NaNO_2_, Merck, Darmstadt, Germany) as standard. Absorption was measured in an automated plate reader (SLT Reader 340 ATTC, SLT-Labinstruments, Achterwehr, Germany) at 540 nm. Values are given as μmol nitrite/mg explant wet weight.

### Quantitative real-time PCR (qPCR)

Relative quantification of different gene transcription levels was performed via real-time PCR. After 72 h of treatment eight explants of one experimental subgroup were pooled, immediately frozen in liquid nitrogen and pulverized. RNA extraction was performed using TRIZOL reagent (1 ml/100 mg wet weight tissue; Invitrogen, Carlsbad, CA, USA) and RNA quality was controlled by measuring OD260/OD280 ratio. Remaining DNA contamination was removed by digestion with DNase (65 °C for 10 min; Promega, Madison, WI, USA). qPCR was performed in a 7500 Fast Real-Time PCR System (Applied Biosystems, Darmstadt, Germany) using the Qiagen QuantiTect SYBR® Green PCR Kit (Qiagen, Hilden, Germany) according to the manufacturer’s instructions: reverse transcription 30 min at 50 °C; PCR initial activation step 15 min at 95 °C; denaturation 15 s at 94 °C; annealing 30 s at 60 °C; extension 30 s at 72 °C; optional: data acquisition 30 s at melting temperature 70 to 78 °C.

All primers were designed using primer express software 3.0.1 (Applied Biosystems, Darmstadt, Germany), ordered at Biomers (Ulm, Germany) and used at a concentration of 0.5 μM (Table 1). Values were taken as duplicates and a comparative quantification (ΔΔCT-method) was performed to analyze the data. Using this method the n-fold expression for the gene of interest is initially adjusted in relation to a reference gene (GAPDH; ΔCT) and secondly normalized to an untreated control group (ΔΔCT). The n-fold expression of the target gene was calculated by the equation 2^- ΔΔCT^ [27].

**Table 1:** List of primers used for real-time qPCR primer

Target	Sequence (5′–3′)
NOS2 sense	CCC-GCA-TGC-AAC-TCC-AA
NOS2 antisense	TCG-TAA-GTC-ATG-AAC-TGC-CAC-TTC
MMP3 sense	CAC-TCA-ACC-GAA-CGT-GAA-GCT
MMP3 antisense	CGT-ACA-GGA-ACT-GAA-TGC-CGT
MMP13 sense	TCT-TGT-TGC-TGC-CCA-TGA-GT
MMP13 antisense	GGC-TTT-TGC-CAG-TGT-AGG-TGT-A
ADAMTS4 sense	GCG-CCC-GCT-TCA-TCA-CTG
ADAMTS4 antisense	TTG-CCG-GGG-AAG-GTC-ACG
COL10A1 sense	CCT-GCC-CGA-GGA-CTT-TGT-AAA
COL10A1 antisense	GAA-AGC-AGA-CAC-AGG-CAT-TCC
GAPDH sense	ATC-AAG-AAG-GTG-GTG-AAG-CAG-G
GAPDH antisense	TGA-GTG-TCG-CTG-TTG-AAG-TCG

### Statistics

Minimum experimental repetition number was three times (detailed n number is given in results section). For each test group different numbers of separate explants were cultured per experiment. In each case explants were harvested from both menisci of both knee joints of one individual animal (*n* = number of independent experiments with individual animals), randomly distributed and separately isolated and measured. All data were tested for normality using the Kolmogorov-Smirnov test. Statistical analysis was performed using Graph Pad prism 5 program (Graph Pad Software Inc., San Diego, CA, USA). One way ANOVA analysis with Bonferroni’s multiple correction was used to compare means among the independent experimental groups. Differences were considered significant if *P* ≤ 0.05. Different letters in the graphs identify experimental groups, which are statistically different. Quantitative data in the text are presented as mean and 95% confidence interval (95% CI).

## Results

### TNF-α dependent cell death was reduced by IL-10

**Fig. 1:**
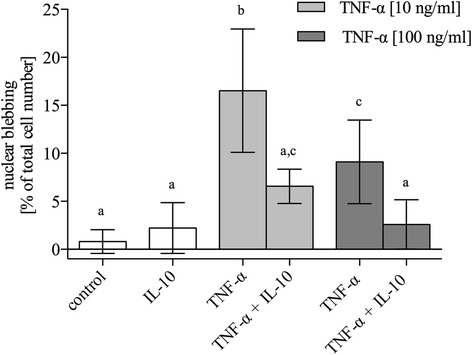
TNF-α dependent cell death was antagonized by IL-10. Meniscus explants were cultured under normal and inflammatory conditions with and without supplemented IL-10 [10 ng/ml]. Explants were stained with Mayer’s hematoxylin in order to visualize cells with nuclear blebbing (NB), an indicator for apoptosis. **a**-**c** indicate clusters of experimental groups which are significantly different from each other with *P* < 0.05 (detailed *p*-values are given in the text). Data are presented as mean ± 95 CI (*n* = 5)

Cell death was assessed by NB indicating an apoptotic cell death after inflammation. Results after statistical analysis are displayed in Fig. 1 (total of 15 disks per test group; *n* = 5). TNF-α [10 ng/ml] induced NB in 16.5% [10.1–22.9] of the cells while TNF-α [100 ng/ml] only reached 9.11% [4.76–13.5], which was a significant increase for both concentrations compared to basic level of apoptotic cells in the untreated control group (0.80% [-0.42–2.04]; *P* < 0.001). Application of IL-10 by itself did not significantly change the basic amount of apoptotic cells in standard culture medium (2.21% [-0.43–4.86]; *P* > 0.05). Addition of IL-10 simultaneously with an inflammatory challenge resulted in an effective and significant reduction of dose-equivalent TNF-α-induced apoptosis by 60.2% (IL-10 + TNF-α [10 ng/ml]: 6.57% [4.78–8.35]; *P* < 0.001) and 71.5% (IL-10 + TNF-α [100 ng/ml]: 2.59% [0.02–5.16]; *P* < 0.05), respectively.

### Production of nitric oxide was attenuated by reduced expression of nitric oxide synthetase 2 (NOS2)

**Fig. 2:**
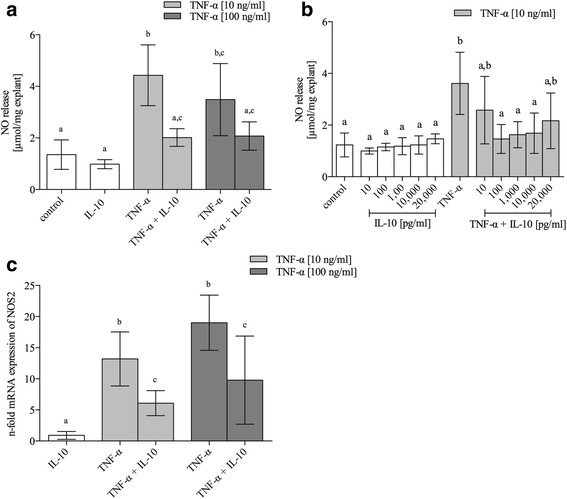
NO release **a**, **b** and NOS2 mRNA expression (**c**) in mature bovine meniscus explants. Explants were cultured under normal culture conditions (control), the influence of IL-10 [10 ng/ml], or after treatment with proinflammatory TNF-α (10 and 100 ng/ml). NO release was measured photometrically by Griess reagent in the culture supernatant (n = 5). For mRNA expression analysis eight disks from the same animal were pooled for mRNA extraction (*n* = 5). Gene expression levels were normalized to that of GAPDH house-keeping gene and then normalized to the non-stimulated control group without compression, which had an expression level = 1. A concentration series for IL-10 is given in figure (**c**) (*n* = 3). **a**-**c** indicate clusters of experimental groups which are significantly different from each other with *P* < 0.05 (detailed *p*-values are given in the text). Data are presented as mean ± 95 CI

For analysis of NO release five independent experiments (*n* = 5, total of 40 disks per test group) with IL-10 [10 ng/ml] were conducted (Fig. 2a). Basic level of NO production was 1.35 μmol/mg [0.78–1.92]. IL-10 reduced the basic rate of NO release by 27.12% without reaching significance (0.98 μmol/mg [0.8–1.16]; *P* > 0.05). Stimulation with TNF-α was accompanied by a significant increase of NO production compared to control group (10 ng/ml: 4.43 μmol/mg [3.25–5.61], *P* < 0.001; 100 ng/ml: 3.49 μmol/mg [2.09–4.88], *P* < 0.001). Addition of IL-10 significantly reduced this up-regulation of NO by 54.63% for TNF-α [10 ng/ml] (2.01 μmol/mg [1.67–2.36], *P* < 0.001) and 40.69% for TNF-α [100 ng/ml] (2.07 μmol/mg [1.52–2.62]; *P* > 0.05). Accordingly, we studied the expression of NO-producing enzyme NOS2 by real time qPCR (*n* = 5). High-dose TNF-α led to a 19-fold increase of NOS2 mRNA expression (19.0 fold [14.6–23.4]), followed by low-dose TNF-α (13.2 fold [8.84–17.5]). IL-10 by itself did not noticeably induce NOS2 mRNA expression (0.9 fold [0.26–1.54]) (Fig. 2c). Reconcilable with the results seen for the NO release, mRNA expression of NOS2 was significantly reduced by addition of IL-10 (TNF-α [10 ng/ml]: 6.08 fold [4.09–8.09], *P* < 0.001; TNF-α [100 ng/ml]: 9.78 fold [2.69–16.9; *P* = 0.004]). Concentration series (*n* = 3, total of 40 disks per test group) with sole IL-10 (dosage ranging from 10 pg/ml to 20 ng/ml) showed a non-significant, but stepwise increase of the basic NO release (Fig. 2b). When administered simultaneously with TNF-α stimulation there was a significant trend for more pronounced reduction of NO with dosages ranging from 100 pg/ml to 10 ng/ml, whereas the lowest and the highest dose reached no significant reduction compared to sole TNF-α. Taken together we can conclude that IL-10 has potential to reduce oxidative stress under inflammatory conditions in meniscus tissue and that this is in part mediated by inhibition of NOS2 transcription.

### TNF-α initiated matrix degeneration was restored by IL-10

**Fig. 3:**
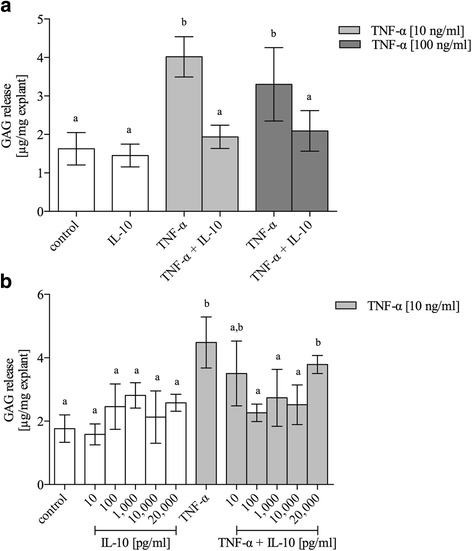
Glycosaminoglycan release in TNF-α stimulated meniscus explants was reduced by IL-10. GAG release was measured in supernatants of mature bovine meniscus explants under normal culture conditions (control), the influence of IL-10 [10 ng/ml], or after treatment with proinflammatory TNF-α (10 and 100 ng/ml). Figure **a** displays results of five independent experiments (*n* = 5) with IL-10 [10 ng/ml]. A concentration series for IL-10 is given in figure **b** (*n* = 3). **a**-**c** indicate clusters of experimental groups which are significantly different from each other with *P* < 0.05 (detailed *p*-values are given in the text). Data are presented as mean ± 95 CI

To evaluate TNF-α dependent matrix degeneration the release of GAG fragments was measured and aggrecanase activity-specific aggrecan neoepitope NITEGE was visualized by immunostaining. Release of GAG fragments was measured from five independent experiments (*n* = 5, total of 40 disks per test group) with IL-10 [10 ng/ml]. The basic release of GAG in controls was 1.63 μg/mm^3^ [1.21–2.05] (Fig. 3a). Incubation with TNF-α induced a significant (both *P* < 0.001) increase of GAG release compared to controls, but was more pronounced for 10 ng/ml (4.01 μg/mm^3^ [3.49–4.54]) than for 100 ng/ml TNF-α (3.30 μg/mm^3^ [2.35–4.26]). Sole IL-10 [10 ng/ml] tended to reduce the basic GAG release compared to the untreated control group without reaching significance (1.45 μg/mm^3^ [1.15–1.75]; *P* > 0.05). The relative decrease of TNF-α-induced GAG release by IL-10 was strongly marked at 10 ng/ml TNF-α (+ IL-10: 1.94 μg/mm^3^ [1.64–2.24], *P* < 0.001) and less marked, yet significant, for 100 ng/ml TNF-α (+ IL-10: 2.09 μg/mm^3^ [1.56–2.62], *P* = 0.013).

We further conducted a concentration series (n = 3, total of 24 disks per test group) with IL-10 dosages ranging from 10 pg/ml to 20 ng/ml (Fig. 3b). Sole IL-10 showed a stepwise, but non-significant, increase of the basic GAG release. Compared to sole TNF-α [10 ng/ml] stimulation (4.17 μg/mm^3^ [3.37–4.97]) the addition of IL-10 resulted in a biphasic GAG reduction with the lowest concentration at 100 pg/ml (+ IL-10 [100 pg/ml]: 2.26 μg/mm^3^ [1.99–2.54], *P* < 0.001).

**Fig. 4:**
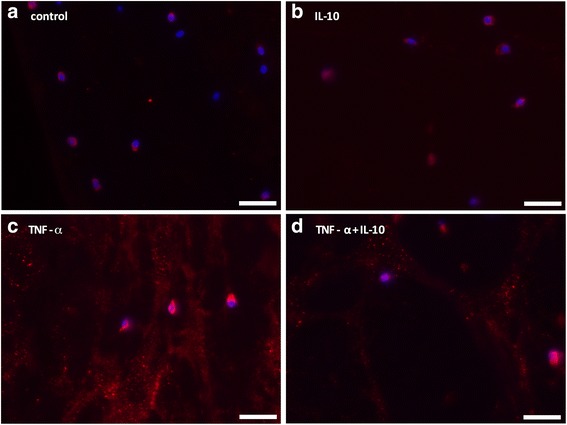
Immunostaining of the aggrecan cleavage product NITEGE of meniscal explants after 3 days of incubation with TNF-α [10 ng/ml] and IL-10 [10 ng/ml]. There was an increase in NITEGE-staining (*red fluorescence*) in TNF-α-treated samples **c** in comparison to control tissues **a** and sole IL-10 stimulation (**b**). IL-10 was able to reduce TNF-α-dependent formation of NITEGE fragments (**d**). Cellular nuclei are counterstained using bisbenzimide (*blue fluorescence*). Scale bar = 50 μm

Immunostaining of the aggrecanase activity-specific aggrecan neoepitope NITEGE showed very low signals in control and IL-10-stimulated tissue (Fig. 4a,b). TNFα-stimulation resulted in a distinct increase in NITEGE positive staining signal, which was decreased by IL-10 (Fig. 4c, d).

### The expression of matrix degrading enzymes and hypertrophic marker collagen type X was reduced by IL-10

**Fig. 5:**
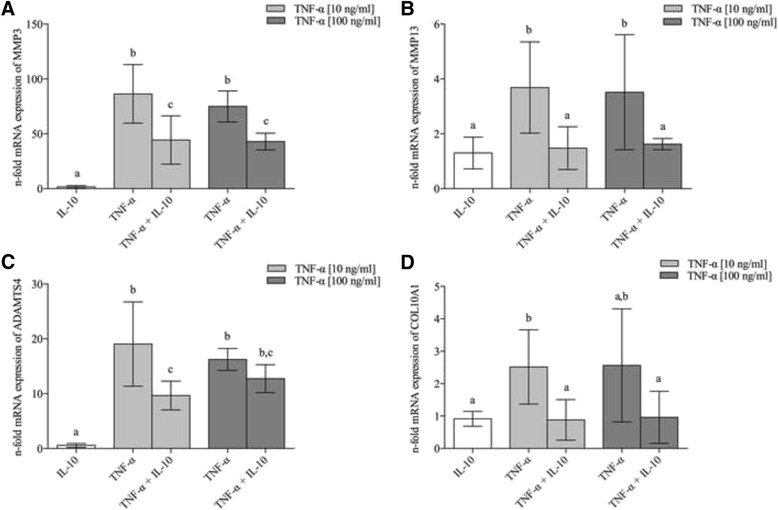
Inflammation-induced gene expression of matrix degrading enzymes and collagen type X was decreased by IL-10. Expression levels of matrix-degrading enzymes were evaluated 72 h after treatment with proinflammatory TNF-α (10 and 100 ng/ml) and anti-inflammatory IL-10 [10 ng/ml]. For each condition eight disks from the same animal were pooled for mRNA extraction (*n* = 5). Expression levels of **a** MMP3, **b** MMP13, **c** ADAMTS4 and **d** COL10A1. Gene expression levels were normalized to that of GAPDH house-keeping gene and then normalized to the non-stimulated control group without compression, which had an expression level = 1. **a**,**b** indicate clusters of experimental groups which are significantly different from each other with *P* < 0.05 (detailed *p*-values are given in the text). Data are presented as mean ± 95 CI

Relative quantification (qPCR) of the mRNA expression levels of different matrix degrading enzymes (Fig. 5a-c) was performed to evaluate the underlying mechanisms of TNF-α induced matrix degradation (*n* = 5). TNF-α stimulation induced a strong increase (both *P* < 0.001 compared to sole IL-10) for MMP3 (TNF-α [10 ng/ml]: 86.2 fold [59.5–113]; TNF-α [100 ng/ml]: 75.0 fold [60.8–89.1]). Less pronounced results were measured for MMP13 (TNF-α [10 ng/ml]: 3.69 fold [2.02–5.35]; *P* = 0.006; TNF-α [100 ng/ml]: 3.52 fold [1.42–5.62]; *P* = 0.007) compared to IL-10-control group, respectively. Again, significant induction (both *P* < 0.001) was measured for the aggrecanase ADAMTS4 (TNF-α [10 ng/ml]: 19.1 fold [11.4–26.7]; TNF-α [100 ng/ml]: 16.2 fold [14.2–18.2]). Highest induction of MMPs was seen for 10 ng/ml TNF-α, which is in line with the total amount of GAG release. IL-10 by itself did not significantly increase the basic transcription rate of all measured enzymes.

For both TNF-α concentrations all matrix-degrading enzymes were significantly reduced when IL-10 was added. With this simultaneously treatment, the expression rate of MMP3 was halved by 48.61% compared to sole TNF-α treatment (TNF-α [10 ng/ml] + IL-10: 44.3 fold [22.3–66.4]; *P* < 0.001; TNF-α [100 ng/ml] + IL-10: 43.0 fold [34.5–50.6]; *P* < 0.05), followed by ADAMTS4 by 49.43% (TNF-α [10 ng/ml] + IL-10: 9.66 fold [7.03–12.3]; *P* < 0.001). Anyway, the reduction of ADAMTS4 for TNF-α [100 ng/ml] + IL-10 (12.7 fold [10.2–15.3]; *P* > 0.05) turned out not to be significant. Even though the results for MMP13 occurred on a lower level, the relative reduction by IL-10 compared to sole TNF-α treatment was most effective by 60% (TNF-α [10 ng/ml] + IL-10: 1.48 fold [0.69–2.26]; *P* < 0.01; TNF-α [100 ng/ml] + IL-10: 1.62 fold [1.42–1.83]; *P* = 0.02).

To evaluate potential anti-hypertrophic properties of IL-10 we further investigated the effect on hypertrophy marker collagen type X (COL10A1). COL10A1 expression was significantly induced by TNF-α compared to sole IL-10 as displayed in Fig. 5d (TNF-α [10 ng/ml]: 2.51 fold [1.37–3.66]; *P* < 0.05; TNF-α [100 ng/ml]: 2.23 fold [-0.31–4.77]; *P* = 0.09). Simultaneous stimulation with IL-10 reduced TNF-α dependent COL10A1 expression by 64.95% (TNF-α [10 ng/ml]: 0.88 fold [0.25–1.51]; *P* < 0.05) and by 56.05% (TNF-α [10 ng/ml]: 0.98 fold [-0.55–2.51]; *P* = 0.23).

## Discussion

The present study evaluated effects of anti-inflammatory IL-10 in an in vitro model of TNF-α-related meniscus degeneration. Our results demonstrate *menisco-protective* effects of IL-10 in terms of cell death, cellular de-differentiation and matrix degeneration. IL-10 prevented early cell death events, which were mainly initiated by TNF-α. It could be shown that addition of IL-10 decreased the TNF-α-induced expression of matrix degrading enzymes, release of GAGs and formation on NITEGE fragments, which suggests that IL-10 prevented crucial mechanisms of meniscus degeneration. In addition, IL-10 reduced the release of NO by decreased expression of NOS2 and therefore interfered with oxidative stress mechanism.

Larsson et al. recently demonstrated a distinct association between elevated joint concentration of TNF- α after meniscectomy and increased risk for progression of radiographic OA [28]. We revealed anti-apoptotic effects of IL-10 in vitro by distinct reduction of cells with NB. Histomorphometric analysis of NB using light microscopy has been confirmed to identify apoptotic cells by electron microscopy previously [25]. Given the low cell density in meniscus tissue, apoptosis is considered as a major pathogenic factor in meniscus tears [29]. Kim et al. reported a particularly significant role of chondrocyte apoptosis in the development of post-traumatic arthritis [30]. Apart from a receptor mediated cell death, the reduction of NO production would be a mechanism in which IL-10 interferes with TNF-α induced cell death.

In addition, IL-10 interacted with inflammation-related matrix degradation and cellular phenotype differentiation. Our results suggest that IL-10 restored the integrity of the meniscal extracellular matrix as it reduced the expression of matrix-degrading enzymes, release of GAG fragments and NITEGE formation. In addition, IL-10 attenuated a hypertrophic phenotype de-differentiation by decreased expression of hypertrophy markers such as collagen type X and MMP13 in autochthonous meniscal cells. Cellular hypertrophy is associated with calcification and fibrocartilage formation, which in turn leads to meniscus and cartilage degeneration [9].

The importance of MMP activity in TNF-α depended proteoglycan degradation in bovine meniscal tissue has been emphasized previously [24]. We focused on ADAMTS4 since Voigt et al. [24] demonstrated increased expression of ADAMTS4 with TNF-α-treatment, whereas ADAMTS5 mRNA expression was low or not detectable. Reduction of ADAMTS4 mRNA by IL-10 was paralleled by decreased detection of NITEGE fragments. Reduction of GAG release and expression of matrix degrading enzymes MMP3, MMP13 and ADAMTS4 indicated protective effects of IL-10. Although extracellular matrix in meniscus tissue only consists of 1% GAG, it has been demonstrated to essentially contribute to compressive and tensile properties in human meniscus [31]. Recently, similar findings have been demonstrated in an in vitro model of mechanically injured articular cartilage. In this model IL-10 was able to inhibit the post-injurious apoptosis and matrix degeneration [32].

Interestingly, the question about the effective dosage for IL-10 under different joint pathologies remains unanswered yet. Our results present first evidence for a distinct dose-response showing more pronounced effects with lower IL-10 dosages. This is in line with a previous study in mechanically injured cartilage, which revealed a similar dosage ranging from 10 to 100 pg/ml IL-10 to be most effective in terms of chondroprotection [32]. The weaker effect of IL-10 at higher doses may have important implications. In this context, the IL-10 signaling pathway may provide a possible explanation. There is evidence that IL-10 auto-regulates its biological effects via a negative feed-back loop involving autocrine stimulation of the IL-10 receptor [33, 34]. In addition, in various immune cells, including synovial macrophages, this feedback-loop has been identified to cause a cellular phenotype switch from classically activated (proinflammatory) to an alternatively activated (anti-inflammatory) phenotype [35, 36]. One could speculate that a self-adjusting mechanism for IL-10 by an autocrine inhibition takes place in fibro-chondrocytes as well and that this is responsible for less pronounced effects with higher IL-10 dosages. Anyway, the concentration of IL-10 in a healthy joint or even after injury has been found to be lot lower than 10 ng/ml [37, 38]. However, at this moment in time a wide range of thematically related studies, that directly applied IL-10, used dosages ranging prom 10 - 300 ng/ml [22, 39–41]. The decrease of efficacy with higher dosages remains interesting and noticeable and should be taken into account by future investigations about IL-10. It remains noticeable that there were no increased effects with higher TNF-α dosages, which could be indicative for a saturation effect since TNF-α effects are mostly receptor-mediated.

A recent study found that fibrocartilage formation and calcification in meniscus tissue is positively correlated with meniscus degeneration and is a predisposing factor for cartilage lesions [6]. This lines up with investigations by Du et al., who demonstrated that abnormal mechanical loading induced cartilage degeneration by accelerating meniscus hypertrophy and mineralization [9]. The results of our study provide evidence that IL-10 inhibits TNF-α-induced hypertrophic de-differentiation, as it reduced the hypertrophy markers collagen type X and MMP13 [42]. We therefore propose that IL-10, especially in lower dosages, has the ability to stabilize the physiological cellular phenotype in inflammatory-challenged meniscus tissue. In addition, Du et al. found post-traumatic cell death and GAG release to be significant pathogenetic mechanisms in their model, which were counteracted by IL-10 under inflammatory as well as in post-injurious conditions [32, 40].

Due to its tissue source and in vitro design our research study has several limitations. Firstly, our study was performed with bovine meniscal explants and it remains questionable if these effects are the same in human OA and PTOA. Secondly, the highly inhomogeneous meniscal tissue, exhibiting regional differences in composition, structure and cellular phenotype, caused a methodical limitation of our results. Our explants were largely punched out of the middle radial third, but there are however explants that have to be attributed rather to the inner or outer radial third, respectively. Since there are zonal differences in matrix turnover and cytokine response [43], we cannot exclude this as a confounding factor in our study. In addition, pretreatment with IL-10 could have induced more pronounced effects, but to our opinion the simultaneous treatment with and without TNF-α and IL-10 reflects the therapeutical approach more closely.

From a translational point of view, the administration of IL-10 and even more important its biologically effective dose in vivo remain challenging questions. IL-10 is a short lasting biological molecule and its effective dosage seems to vary depending on different joint pathologies. Targeted gene therapy might offer an effective approach, which allows a disease-regulated overexpression of IL-10 within the damaged joint [44, 45]. Another approach could be the injection into the rupture margins during arthroscopic meniscus suture or interposition of an IL-10-soaked scaffold. To the best of our knowledge there are no studies that examined the ability of IL-10 to penetrate meniscal tissue by diffusion yet. Since we used no monoculture, but a tissue culture, IL-10 must have had the ability to penetrate the explant tissue and reach the fibro-chondrocytes, since we can present significant effects by administration of IL-10.

Further studies should examine the long-term effects of IL-10 in terms of tissue remodeling and cell differentiation of fibro-chondrocytes. In this regard the effective IL-10 dose in chronically inflamed meniscus tissue seems to be of particular importance.

## Conclusion

Taken together we conclude that anti-inflammatory IL-10 prevented crucial mechanisms of inflammation-related meniscus degeneration. TNF-α dependent cell death and extracellular matrix degeneration were decreased by IL-10, which is indicative for potential menisco-protective properties in clinical situations of meniscus degeneration or meniscus-related sport injuries.

## Abbreviations

ADAMTS4/5: A disintegrin and metalloproteinase with thrombospondin motifs 4 or 5
CI: Confidence interval
COL10A1: Gene encoding collagen alpha-1(X) chain
GAG: Glycosaminoglycans
GAPDH: Gene encoding glyceraldehyde 3-phosphate dehydrogenase (reference gene) IL-10, Interleukin 10
MMP: Matrix Metalloproteinase
MMP3/13: Gene encoding matrix metalloproteinase 3 or 13
mRNA: messenger RNA
NB: Nuclear blebbing
NITEGE: Neoepitope sequence generated by aggrecanases-mediated cleavage at Glu373- Ala374 of aggrecan core proteins
NO: Nitric oxide
NOS2: Gene encoding nitric oxide synthase 2
OA: Osteoarthritis
PTOA: Post traumatic osteoarthritis
qPCR: Quantitative polymerase chain reaction
SOX9: Gene encoding a member of the SOX (SRY-related HMG-box) family of transcription factors
TNF-α: Tumor necrosis factor alpha
